# Evaluating the factor structure, item analyses, and internal consistency of hospital anxiety and depression scale in Iranian infertile patients

**Published:** 2017-05

**Authors:** Payam Amini, Saman Maroufizadeh, Reza Omani Samani

**Affiliations:** *Department of Epidemiology and Reproductive Health, Reproductive Epidemiology Research Center, Royan Institute for Reproductive Biomedicine, ACECR, Tehran, Iran.*

**Keywords:** Anxiety, Depression, Factor analysis, Infertility

## Abstract

**Background:**

The hospital anxiety and depression scale (HADS) is a common screening tool designed to measure the level of anxiety and depression in different factor structures and has been extensively used in non-psychiatric populations and individuals experiencing fertility problems.

**Objective:**

The aims of this study were to evaluate the factor structure, item analyses, and internal consistency of HADS in Iranian infertile patients.

**Materials and Methods:**

This cross-sectional study included 651 infertile patients (248 men and 403 women) referred to a referral infertility Center in Tehran, Iran between January 2014 and January 2015. Confirmatory factor analysis was used to determine the underlying factor structure of the HADS among one, two, and three-factor models. Several goodness of fit indices were utilized such as comparative, normed and goodness of fit indices, Akaike information criterion, and the root mean squared error of approximation. In addition to HADS, the Satisfaction with Life Scale questionnaires as well as demographic and clinical information were administered to all patients.

**Results:**

The goodness of fit indices through CFAs exposed that three and one-factor model provided the best and worst fit to the total, male and female datasets compared to the other factor structure models for the infertile patients. The Cronbach’s alpha for anxiety and depression subscales were 0.866 and 0.753 respectively. The HADS subscales significantly correlated with SWLS, indicating an acceptable convergent validity.

**Conclusion:**

The HADS was found to be a three-factor structure screening instrument in the field of infertility.

## Introduction

The Hospital anxiety and depression scale (HADS) is a self-report screening tool to measure psychological distress which is widely and increasingly used. The HADS is a 14-itme instrument comprising which the first and the latter 7 items measure the anxiety and depression respectively and from separate anxiety and depression scores are calculated (1). This instrument has been assessed using several psychiatric and primary care populations which exclude symptoms arise from the somatic aspects of the disease. The most advantage of HADS is the conciseness which allows one to utilize it for clinical, medical, and research settings (2). Despite its well-proved reliability and validity, several studies resulted in inconsistent factor structures for the HADS. For example, some studies showed that this instrument is formed as a single structure, a two-factor structure, a three-factor one and four factors (3-10). However, the resulted extra factors were considerably associated with anxiety and depression (7). 

The HADS was basically a cancer research device but lots of other medical areas use it to report the depressive symptoms such as Covic *et al. *that provided further support for high prevalence of depression and anxiety in rheumatoid arthritis, Cosco *et al* in investigating patients with cardiovascular disease and Barth and Martin who determined to whether the three-factor structure of the HADS has the same psychometric properties in German patients presenting with CHD (11-13). Moreover, it can be of importance to assert if the HADS is usable in non-psychiatric populations (2). Infertility is one of the universal concerns in adults specially those whose plans include children. Infertility is a global public health issue and affects approximately 10-15% of reproductive-aged couples worldwide (14).

Although the global trend in infertility has not been changed over the recent 20 years, the number of couples affected by infertility increased from 42 million in 1990 to 48.5 million in 2010 (15). Undeniably, infertility causes lots of distressing and anxious experiences such as loss of self-esteem, depression, frustration, emotional and sexual distress and marital problems (16-20). Lawson *et al*. showed that psychologic consultation before treatment must be prepared for infertile patients to identify situation and anxiety symptoms (21). Fassino* et al* investigated the association of depression, anxiety and expressed emotional patterns to infertility (22). Infertility and its treatment have deleterious effects on person's quality of life and subjective well-being (23-25).

Some studies have investigated the association of factors with the anxiety and depression of female infertility patients using the HADS while no investigation of the factor structure of translated HADS in Iranian infertile patients has been performed (26). According to the adverse effects of stress, anxiety, and depression in infertile patients, it could be a good rationale for applying the HADS. Based on the predictive characteristics of the HADS in the clinical oncology setting, finding the best structure of the HADS for infertile patients can improve the predictions and prevent harmful events (5). 

Therefore, the present study aims to examine the factor structure of the Persian version of the HADS in infertile patients using several performed models presented in table I.

## Materials and methods

**Patients and study design**

In this cross-sectional study, the participants were recruited using random sampling method between January 2014 and January 2015 from the infertility clinic at Royan Institute, a referral center in Tehran, Iran (27). Couples suffering from infertility come to this clinic, not only from the capital of Iran but also from all around the country. The sample size was calculated according to a general rule of thumb for factor analysis “at least 500 cases” (28, 29). The inclusion criteria for this study were as follows: (a) age >18 yr; (b) experiencing fertility problems; (c) ability to read and write in Persian. The exclusion criterion was an unwillingness to participate in this study. Moreover, incomplete questionnaires were excluded. In total, 651 patients (248 men and 403 women) agreed to participate and completely filled out the instruments.

**Demographic and clinical characteristics**

Demographic and clinical characteristics included in this study were age, duration of infertility, sex, educational level, the cause of infertility, and the history of abortion.

**Hospital anxiety and depression scale (HADS)**

The HADS is a widely used self-report tool designed as a brief assessment of both anxiety and depression in non-psychiatric populations. The HADS comprises only 14-items consisting of two subscales of seven items that assess levels of anxiety (HADS-A) and depression (HADS-D). Each item is scored on a 4-point Likert scale ranging from 0-3, with a score range of 0-21 for both subscales. Higher scores indicate a greater anxiety and depression state. In this study, the Persian version of HADS translated by Montazeri *et al *was used (30).

**Satisfaction with life scale (SWLS)**

The SWLS is a short 5-item instrument developed by Diener *et al* in 1985 that assess satisfaction with the respondent’s life as a whole. Items are rated on a 7-point Likert scale, ranging from 1 (strongly disagree) to 7 (strongly agree). Total scores range from 5 to 35, with higher scores indicating greater life satisfaction. The Persian version of SWLS has shown good psychometric properties (31). The Cronbach’s alpha coefficient of SWLS in the present study was 0.855.

**Ethical consideration**

Ethical approval was obtained from the Ethics Committee of Royan Institute, Tehran, Iran. All participants were informed about the study’s scope and objectives, and the confidentiality of the data. Verbal informed consent was obtained from all participants prior to data collection.

**Statistical analysis**

**Confirmatory factor analysis**

In factor analysis, several variables (here such as questions in the HADS questionnaire) are formed as linear combinations of a few random variables which are called factors (here such as anxiety and depression). If a questionnaire is consist of “*p*” correlated questions, then the basic dimensionality of the questionnaire is less than “*p*”. Factor analysis reduces the redundancy among the questions by using a smaller number of factors. Confirmatory factor analysis is used to confirm or reject a predetermined factor structure. The parameter estimation is carried out using a correlation matrix of questions. 

Using statistical software LISREL version 8.80, the confirmatory factor analysis was performed. Several models of the HADS was evaluated such as Zigmond and Snaith’s, Moorey *et al *which both are two factor models, Razavi* et al* as a single factor model and Dunber *et al*, Friedman *et al*, Caci *et al*, Leung *et al*, Brandberg *et al, *and Kaur *et al *as three-factor structure models (3, 7-9, 32-37). The outperformed models were determined using several goodness of fit indices including the comparative fit index (CFI greater than 0.90), the Akaike information criterion (AIC the smaller the better), the normed fit index (NFI greater than 0.90), the goodness of fit index (GFI greater than 0.90) and the root mean squared error of approximation (RMSEA less than 0.08) and ^2^ with the degree of freedom (df) where a less than three ^2^/df indicated a good fit (38, 39). Moreover, the Chi-square goodness of fit test was used which a significant one concludes non-sufficient model (4, 40). 

## Results

**Patients characteristics**

In total, 651 patients (248 men and 403 women) met the eligible criteria and participated in the study. Table II shows the demographic and clinical characteristics of the sample. The mean age of participants was 31.16 years (SD=5.87, range 18-63) and the mean duration of infertility was 5.16 years (SD=3.77, range 1-30). The majority of the patients had male factor infertility (41.0%), university education (39.8%), and no history of abortion (84.6%).

The mean HADS-A subscale score was 7.73 (SD=4.44, range 0-21) and the mean HADS-D subscale score was 5.96 (SD=3.82, range 0-21). The mean HADS-A and HADS-B subscale score for males were 6.13 (SD=3.99, range 0-18) and 5.64 (SD=5.64, range 0-18) and for females 8.72 (SD=4.42, range 0-21) and 6.16 (SD=3.78, range 0-21) respectively. Using Snaith and Zigmond's cut-off criteria of HADS-A and HADS-D scores of 8 or over, 315 participants (48.4%) demonstrated possible clinically relevant levels of anxiety and 218 participants (33.5%) possible clinically relevant levels of depression (32). Adopting Snaith and Zigmond's higher threshold for the sensitivity of HADS-A and HADS-D scores of 11 or over, 173 participants (26.6%) demonstrated probable clinically relevant levels of anxiety and 87 participants (13.4%) probable clinically relevant levels of depression.

**Confirmatory factor analysis**

The 10 mentioned models were tested and compared using the goodness of fit. According to the chi-square index, none of the models explained the total variance. For the total, male and female data, the Dunber *et al* model was determined as the best performing factor structure based on the GFI, NFI, and CFI greater than 0.90, the RMSEA less than 0.06 and the least AIC. Moreover, the worst performance belonged to Razavi *et al* model for the three sets of the data. The order of best fitting models is not the same for the total, male and female data set. Details are shown in table III. Figures one to three show the best-fitted models for total data, female data and male data, respectively.

**Reliability and item analysis**

Cronbach’s alpha coefficient of the HADS-A and HADS-D subscales were 0.866 and 0.753 respectively, exceeding Kline's criterion for acceptable instrument internal consistency. As seen in table V, these values did not improve if an item was deleted from the subscale. All corrected item-total correlations were greater than the acceptable cut-off of 0.3 indicating each item was related to its total subscale. The inter-item correlations of HADS-A and HADS-D (data not shown) were also acceptable within the range of 0.382-0.547 and 0.215-0.457 respectively. The mean and standard deviation for each item are also presented in table IV.

**Convergent validity**

Convergent validity of the HADS was assessed by examining correlations with SWLS. As expected, both HADS-A and HADS-D subscales showed significant negative correlations with SWLS score (r=-0.357 and r=-0.429, respectively).

**Table I:** Characteristics of each factor model tested

**Model**	**No. of factors**	**Population**	**Sample size**	**Extraction method**	**Factor 1**	**Factor 2**	**Factor 3**
Zigmond-Snaith (30)	2	Medical	100	None	1,3,5,7,9,11,13	2,4,6,8,10,12,14	----
Moorey *et al* (31)	2	Cancer	568	PCA a	1,3,5,9,11,13	2,4,6,7,8,10,12,14	----
Dunbar *et al* (9)	3	Non-clinical	2547	CFA b	1,5,7,11	2,4,6,7,8,10,12,14	3,9,13
Friedman *et al* (32)	3	Depressed	2669	PCA	1,7,11	2,4,6,8,10,12,14	3,5,9,13
Razavi *et al* (3)	1	Cancer	210	PCA	All items	----	----
Caci *et al* (7)	3	Non-clinical	195	PCA	7, 11, 14	2, 4, 6, 8, 10, 12	1, 3, 5, 9, 13
Caci *et al* (7)#	3	Non-clinical	195	PCA	7, 11, 14	2, 4, 6, 8, 12	1, 3, 5, 9, 13
Leung *et al* (33)	3	Non-clinical	141	PCA	3, 8, 10, 11	2, 4, 6, 7, 12, 14	1, 5, 9, 13
Brandberg *et al* (35)	3	Cancer	273	PCA	1, 7, 11, 14	2, 4, 6, 8, 10, 12	3, 5, 9, 13
Kaur *et al* (34)	3	CAD	189	PCA	1,3,5,7,9,11,13	2, 4, 6, 14	8, 10, 12

a Principle component analysis,

b Confirmatory factor analysis,

# Item 10 is removed

**Table II:** Demographic and clinical characteristics of the infertile patients (n=651)

**Variables**		**Mean±SD or n(%)**
Age (years)		31.16±5.87
Duration of infertility (years)		5.16±3.77
Sex		
	Male	248 (38.1)			
	Female	403 (61.9)			
Cause of infertility		
	Male factor	267 (41.0)			
	Female factor	181 (27.8)			
	Both	72 (11.1)			
	Unexplained	131 (20.1)			
Educational level		
	Primary	158 (24.3)		
	Secondary	234 (35.9)		
	University	259 (39.8)		
History of abortion		
	No	551 (84.6)	
	Yes	100 (15.4)	

**Table III:** The results and the comparison of factor structure of the HADS using 10 models

**Sample**	**Models**	^2^** (df**^a^**)**	^2^** /df**	**RMSEA** b	**CFI** c	**NFI** d	**GFI** e	**AIC** f	**The order of best fitting**
Total									
	Dunbar *et al* (9)	192.55 (72)	2.67	0.051	0.98	0.97	0.96	258.55	1
	Friedman *et al* (32)	221.29 (74)	2.99	0.055	0.98	0.97	0.95	283.29	2
	Caci *et al* (7) #	238.16 (62)	3.84	0.066	0.97	0.96	0.95	296.16	3
	Moorey *et al* (31)	239.14 (76)	3.14	0.057	0.98	0.96	0.95	297.14	4
	Zigmond-Snaith (30)	246.11 (76)	3.23	0.059	0.98	0.96	0.95	304.11	5
	Kaur *et al* (34)	238.96 (74)	3.22	0.060	0.98	0.96	0.95	307.87	6
	Caci *et al* (7)	261.27 (74)	3.53	0.062	0.97	0.96	0.95	323.27	7
	Brandberg *et al* (35)	284.45 (74)	3.84	0.066	0.97	0.96	0.94	346.45	8
	Leung *et al* (33)	329.61 (74)	4.45	0.073	0.97	0.95	0.93	391.61	9
	Razavi *et al* (3)	684.12 (77)	8.88	0.112	0.94	0.93	0.87	740.12	10
Male									
	Dunbar *et al* (9)	136.86 (72)	1.90	0.060	0.97	0.93	0.93	202.86	1
	Friedman *et al* (32)	153.74 (74)	2.07	0.066	0.96	0.93	0.92	215.74	2
	Moorey *et al* (31)	163.27 (76)	2.14	0.068	0.96	0.92	0.91	221.27	3
	Kaur *et al* (34)	163.95 (74)	2.21	0.069	0.96	0.92	0.92	222.10	4
	Zigmond-Snaith (30)	165.34 (76)	2.17	0.069	0.96	0.92	0.91	223.34	5
	Caci *et al* (7) #	170.61 (62)	2.75	0.084	0.94	0.91	0.90	228.61	6
	Brandberg *et al* (35)	190.31 (74)	2.57	0.080	0.94	0.91	0.90	252.31	7
	Caci *et al* (7)	191.89 (74)	2.59	0.080	0.94	0.91	0.90	253.89	8
	Leung *et al* (33)	212.05 (74)	2.86	0.087	0.94	0.91	0.89	274.05	9
	Razavi *et al* (3)	405.91 (77)	5.27	0.130	0.89	0.86	0.81	461.91	10
Female									
	Dunbar *et al* (9)	133.32 (72)	1.85	0.046	0.99	0.97	0.95	199.32	1
	Caci *et al* (7) #	142.97 (62)	2.30	0.057	0.98	0.97	0.95	200.97	2
	Friedman *et al* (32)	151.08 (74)	2.04	0.051	0.98	0.97	0.95	213.08	3
	Moorey *et al* (31)	155.25 (76)	2.04	0.051	0.98	0.96	0.95	213.25	4
	Caci *et al* (7)	155.49 (74)	3.30	0.052	0.98	0.97	0.95	217.49	5
	Zigmond-Snaith (30)	160.22 (76)	2.18	0.053	0.98	0.96	0.95	218.22	6
	Kaur *et al* (34)	153.10 (74)	2.06	0.053	0.98	0.96	0.95	219.92	7
	Brandberg *et al* (35)	175.18 (74)	2.36	0.058	0.98	0.96	0.94	237.18	8
	Leung *et al* (33)	192.69 (74)	2.60	0.063	0.97	0.96	0.94	254.69	9
	Razavi *et al* (3)	335.88 (77)	4.36	0.091	0.95	0.94	0.89	391.88	10

All chi-square analyses were significant (alpha=0.05),

# : Item 10 was removed

a degree of freedom

b The root mean square error of approximation

c The comparative fit index

d normed fit-index

e goodness of fit index

f Akaike information criterion

**Table 4:** Items Wording and Descriptive Statistics of the HADS

**Item**	**Mean**	**SD** ^a^	**Corrected item total correlation**	**Alpha if item deleted**
HADS^b^-Anxiety				
(A1) I feel tense or wound up	1.33	0.84	0.659	0.844
(A3) I get a sort of frightened feeling as if something awful is about to happen	1.29	0.98	0.656	0.845
(A5) Worrying thoughts go through my mind	0.99	0.96	0.674	0.842
(A7) I can sit at ease and feel relaxed	1.22	0.79	0.551	0.858
(A9) I get a sort of frightened feeling like 'butterflies' in the stomach	1.15	0.77	0.652	0.845
(A11) I feel restless as if I have to be on the move	1.02	0.83	0.636	0.847
(A13) I get sudden feelings of panic	0.73	0.79	0.650	0.845
HADS-Depression				
(D2) I still enjoy the things I used to enjoy	1.01	0.93	0.450	0.728
(D4) I can laugh and see the funny side of things	0.73	0.81	0.510	0.715
(D6) I feel cheerful	0.93	0.80	0.533	0.710
(D8) I feel as if I am slowed down	1.14	0.82	0.423	0.733
(D10) I have lost interest in my appearance	0.85	0.93	0.430	0.733
(D12) I look forward with enjoyment to things	0.51	0.84	0.491	0.719
(D14) I can enjoy a good book or TV program	0.78	0.88	0.459	0.725

a : Standard deviation,

b : Hospital Anxiety and depression scale

**Figure 1:**
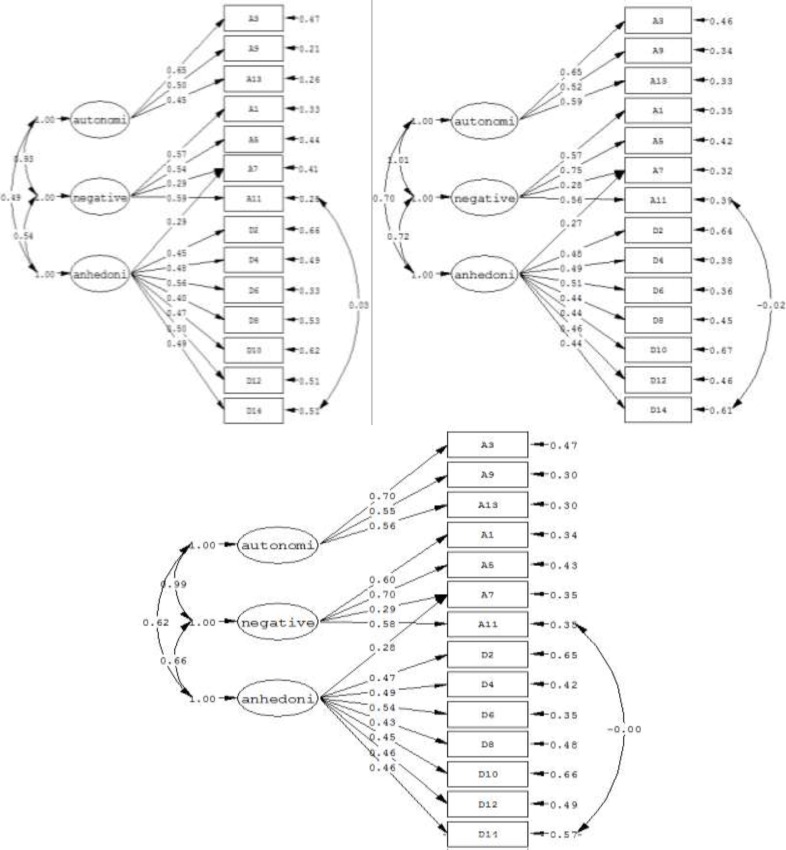
The resulted factor structure of Dunber*et al.’*s model on the data (Total (lower), Male (up-left), Female (up-right))

## Discussion

The purpose of this study was to evaluate the factor structure of the HADS on Iranian infertile patients. This study result showed a high mean level of anxiety 7.73 (SD=4.44) and depression 5.96 (SD=3.82) among infertile patients. The mean level of anxiety in males was significantly lower than females while depression was statistically the same. Biringer *et al* at 2015 demonstrated the mean level of anxiety and depression as 4.5 (SD=3.37) and 2.6 (SD=2.71) respectively which showed a significant association between anxiety/depression and infertility (41). 

Kahyaoglu and Kaplan assessed the Quality of life (e.g. calm and joy) in women with infertility via the FertiQoL and the HADS where a negative association was found between the quality of life and the HADS-A and HADS-D subscale scores (42). In a research dealing with psychiatric morbidity in infertile patients in a tertiary care setup, Verma *et al* used HADS and showed that 56.4% and 68.9% of the females are suffering from depression and anxiety and depression both (43). 

The internal reliability of the HADS-A and HADS-D subscales were statistically acceptable where are totally consistent with studies dealing with reliability (44). The HADS-A and HADS-D were statistically positively correlated as can be found in previous studies (45). A good consistency of the HADS-A and HADS-D in addition to the acceptable reliability makes HADS to be a valid and reliable screening instrument of anxiety and depression and it can be utilized in patients with infertility. 

The CFA findings in this study showed that the HADS is formed through three factors in the infertile patients. Although the chi-square was statistically significant in all of the performed models, the proportion of this statistic to its degree of freedom- which can be used as a comparing tool among performed models in addition to the sufficiency of the model- revealed that Dunber *et al* and Friedman *et al* three-factor structured models were the best and the second best-fitted models respectively in the total dataset. This statistic was less than three for the models fitting the male dataset while the same result was outputted for the models fitting the female dataset except for the Razavi *et al* and Caci *et al* models. Dunber *et al* three-factor model provided the best fit to the infertility total data, males, and females datasets according to several goodness of fit indices such as GFI, NGI, CFI, RMSEA, and AIC (9). 

In the study by Dunbar *et al* (2000), several models were compared to Clark and Watson’s (1991) tripartite theory of anxiety and depression using confirmatory factor analyses (9). The second best model was the Friedman *et al* for the total data and male dataset while the Caci *et al* model ( item 10 is removed from the HADS) fitted the female dataset as the second best model. The second best model was not the same for the female datasets comparing to the male and the total datasets but all are three-factor structure models. Perhaps the three-factor structure of the HADS is related to its fundamental structure. Several studies resulted in a three-factor structure of the HADS with different and unrelated clinical presentations. The resulted structure can be found in the findings of lots of clinical and non-clinical studies (6, 13, 37, 46-48). 

The third best fit to the total and female datasets was performed by the three-factor Caci *et al *and Friedman *et al* models while the two-factor structure model, Moorey *et al*, fitted the male dataset as the best third model (7, 33, 34). The two-factor model, Moorey *et al* applied an exploratory factor analysis of the HADS in 568 cancer patients and supported the use of the separate subscales of the HAD in studies of emotional disturbance in cancer patients (33). The two-factor structure model, Zigmond and Snaith was the fifth best model for the total and male datasets and the sixth best one for the female dataset (32). Zigmond and Snaith applied the HADS on 50 medical patients and found the two-factor as a reliable structure followed some other studies (32, 45, 49-51). The one-factor model was presented by the Razavi *et al* screening for adjustment disorders and major depressive disorders in cancer in-patients which demonstrated the poorest fit to the datasets (3). 

However, in our study, all the performed models had the statistically acceptable goodness of fit indices such as GFI, NFI, and CFI to the data, the Dunber *et al* was the best and the one-factor model, the Razavi *et al* was the worst fitted model. The best fit was provided by Dunber *et al* three-factor model where autonomic anxiety, negative affectivity, and anhedonic depression were the three factors forming the structure (34). The two-factor structures also were formed through two subscales of depression and anxiety and the one-factor one uses all 14 items. 

This study has evaluated the factor structure of the HADS in an infertile population. Several studies used the HADS to report the percentage of anxious and depressed infertile patients but not the structure evaluation of the HADS. Anderson *et al *examined the emotional distress and infertility-related concerns in couples and determined the changes over time (52). Matsubayashi *et al *assessed the increased depression and anxiety in infertile Japanese women, Glover *et al* evaluated the development of the fertility adjustment scale, Slade *et al *investigated the relationship between perceived stigma, disclosure patterns, support and distress in new attendees at an infertility clinic, and Fido and Zahid assessed the coping with infertility among Kuwaiti women (53-56).

Based on the results of this study, the HADS can be used as a useful screening instrument for infertility as a three-factor structure. However, further research is needed in this area to determine if the resulted three-factor structure is reliable in other populations. According to our result in the case of infertility, the two-factor structure fits the poorest among other structures and the one-structure is poorer than the three-factor structure. Hence, it is recommended that the total and two subscale score should not be applied in this clinical context.

## Conclusion

The HADS was found to be a three-factor structure screening instrument in the field of infertility. Besides, the other factor structures (one and two) fitted the data statistically well but not as much as three-factor structure. This can be evaluated by further studies in this context to determine any noteworthy clinical result in a three-factor structure scoring.
